# Spontaneous direct carotid-cavernous fistula with acute visual loss in an elderly patient: case report and review of the literature

**DOI:** 10.1093/omcr/omac086

**Published:** 2022-08-18

**Authors:** Can-Min Zhu, Wei Zeng, Xian Zhang, Qiang Li, Mei Zhang, Chang-Chang, Di-Li Wang

**Affiliations:** Department of Neurology, The First People’s Hospital of Jiangxia District, Wuhan, Hubei 430050, China; Department of Neurology, The Fifth Hospital of Wuhan, Hubei 430050, China; Department of Neurology, The Fifth Hospital of Wuhan, Hubei 430050, China; Department of Neurology, The Fifth Hospital of Wuhan, Hubei 430050, China; Department of Neurology, The Fifth Hospital of Wuhan, Hubei 430050, China; Department of Neurology, The First People’s Hospital of Jiangxia District, Wuhan, Hubei 430050, China; Department of Neurology, The First People’s Hospital of Jiangxia District, Wuhan, Hubei 430050, China

## Abstract

Carotid-cavernous fistulas are acquired vascular lesions, representing abnormal connections caused by a direct shunt or dural branches of the internal carotid artery and/or the external carotid artery connected to the cavernous sinus. We present an interesting, rare case of a spontaneous fistula with acute vision loss in an older patient. The patient was diagnosed with a direct carotid-cavernous fistula based on the clinical and radiological findings. We concluded that early diagnosis and etiological treatment could improve the prognosis of this type of carotid-cavernous fistula.

## INTRODUCTION

Spontaneous carotid-cavernous fistula (CCF) is characterized by the presence of a low flow fistula, low morbidity and complex clinical symptoms [1]. It is commonly insidious and easily misdiagnosed [1]. In this case, the patient was an older woman who exhibited acute visual loss and no other ocular nerve damage. This case report documents her rare clinical manifestations, the completed imaging studies and an uncommon treatment protocol.

## CASE REPORT

A 66-year female was admitted with ‘right eye socket distension for one month and sudden blindness for one day.’ One month previously, the patient felt her right eye become slightly swollen and painful but did not receive any treatment. The symptoms occurred repeatedly. The pain in her right eye socket increased slightly and persisted for more prolonged times after three weeks. The vision in her right eye was blurred, and she experienced double-vision one day before being seen at the ophthalmology center. The patient was informed that her eyes were normal, and she was referred to our department of neurology. The patient had no history of hypertension, diabetes, heart disease, stroke, surgical procedures or injury.

The patient's physical examination revealed the following information. Her blood pressure was 184/ 86 mmHg, the left pupil diameter was 3 mm as measured by a pupilometer, the direct light reflection of the left eye was sensitive, and the indirect light reflection was absent. Her vision in her left eye was normal after receiving optometry, according to International Standard Vision Tables. Her right pupil diameter was 4.5 mm. The direct and indirect light reflections of the right eye were absent. The vision in the patient’s right eye was completely lost. The right fundus oculi vein was filled. Both eyeballs did not exhibit conjunctival congestion or exophthalmos and could move freely. The other components of the neurological examination were normal. After being hospitalized, the patient was determined to have elevated blood glucose and lipids.

**Figure 1:**
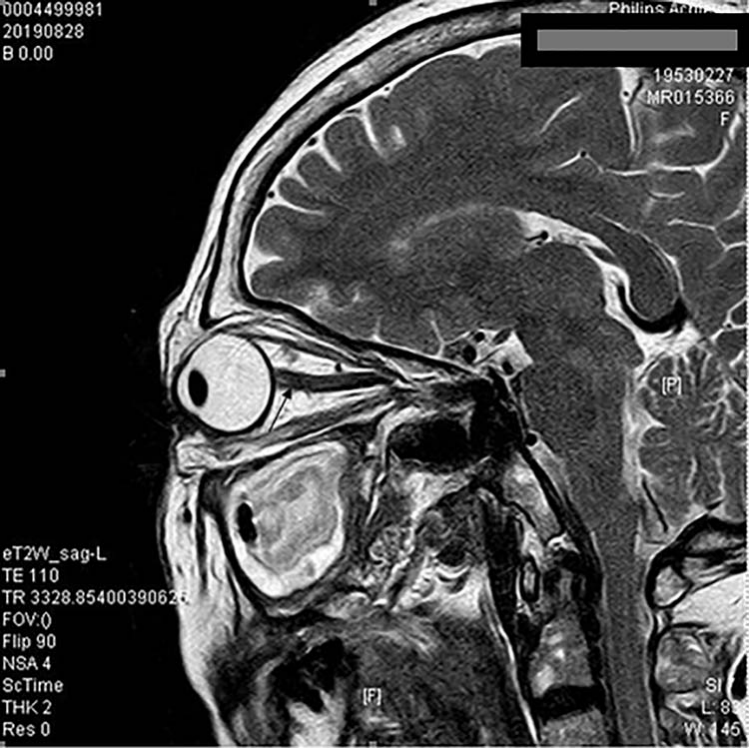
Orbital MRI showed local edema of the right optic nerve.

**Figure 2:**
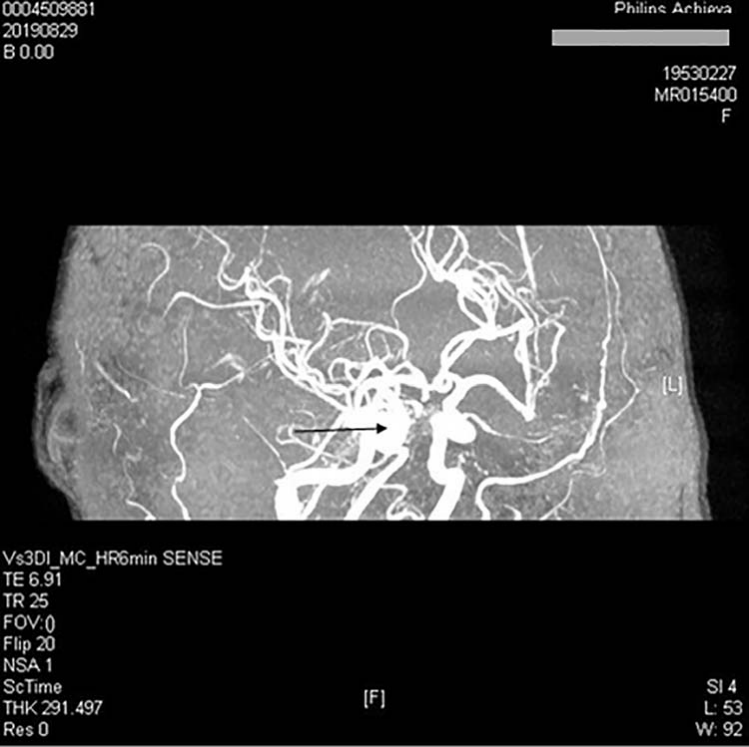
Intracranial MRA showed the right cavernous sinus’s blood vessels were thickened and confusion.

**Figure 3:**
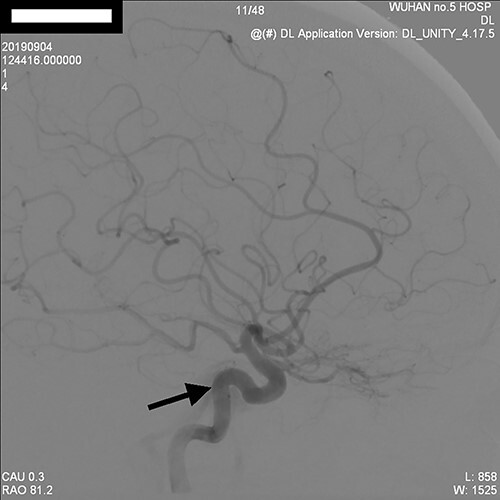
Selective right internal carotid arteriogram (positive view) showed a small fistula between dural CCF and peritoneal pituitary artery, and the sinus drainage was through the lower sinus rock.

**Figure 4:**
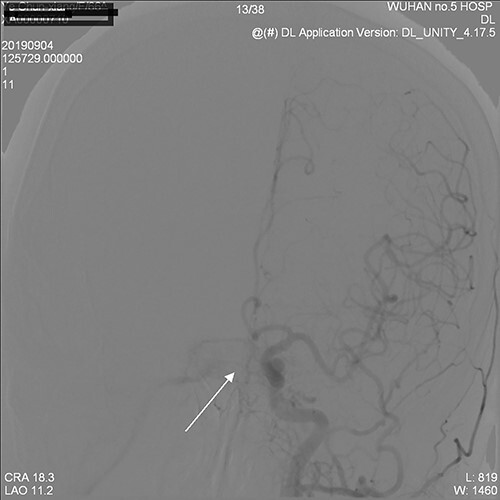
Bilateral cavernous sinus was visualized in the middle arterial period.

The computed tomography (CT) scan of the head for this patient was normal. The orbital magnetic resonance imaging (MRI) revealed localized edema of the right optic nerve (Fig. 1). The intracranial magnetic resonance angiography (MRA) indicated that the right cavernous sinus’s blood vessels were thickened and tortuous (Fig. 2). The cerebral digital subtraction angiography (DSA) showed a small fistula between the meningo-pituitary trunk and the cavernous sinus of the internal carotid artery (ICA), which was drained by the inferior petrosal sinus, and bilateral cavernous sinuses were seen in the mid-arterial location (Figs 3 and 4).

The patient was treated with methylprednisolone 120 mg intravenously once per day. After three days, the methylprednisolone was decreased to 50 mg per day, then stopped after seven days. Her vision gradually improved from no light perception to a 100 cm index (0.1 × 1/5 = 0.02). After being discharged, the patient received finger compression of the right common carotid artery. The compression of the right common carotid artery was performed using two or three fingers placed on the common carotid associated with the lesion until the superficial temporal artery was not visible. Each compression initially lasted no longer than five minutes. This procedure was repeated once after an hour. Gradually, the compression time was extended until the continuous compression time reached 20 to 30 minutes. During the compression, care was taken that the patient did not experience any dizziness, black eyes, contralateral limb weakness or numbness, or other adverse symptoms. Two weeks later, the patient’s vision was improved to three meters (0.1 × 3/5 = 0.06).

## DISCUSSION

The incidence of CCF is quite low. The most common cause is trauma, and spontaneous CCF is exceedingly rare [1]. This patient presented with chronic symptoms accompanied by acute aggravation. She experienced only eye distention and sudden vision loss without other neurological deficits, which is highly unusual. The patient was confirmed to have a cavernous fistula based on the intracranial MRA and cerebral DSA examinations [2].

CCF typically exhibits bulbar conjunctival congestion, edema, exophthalmos and other symptoms. The II, III, IV and VI cranial nerves are typically involved to varying degrees. CCF is easily misdiagnosed as conjunctivitis, glaucoma, retrobulbar abscess and other conditions [3]. The pathogenesis of spontaneous CCF is unknown. However, possible causes are thought to include [4]: (1) endocrine changes; (2) congenital vascular abnormalities, such as dural arteriovenous malformations and aneurysms in the region of the cavernous sinus; (3) changes in vessel fragility and degeneration of arterial walls, such as atherosclerosis. Middle-aged and older persons with hypertension, diabetes, abnormal lipid metabolism or other atherosclerotic risk factors that increase vascular wall fragility are at higher risk for CCF. When blood pressure is increased or accompanied by an aneurysm that causes thinning of the blood vessel wall, the blood vessel may rupture, forming a cavernous fistula.

It is highly uncommon in clinical practice to observe only the optic nerve affected in CCF and no other cranial nerves (i.e. III, IV and VI), and no conjunctival congestion, pulsatile exophthalmos or other symptoms [5]. In this case, the cause for the reduced number of symptoms might be related to the low position of the fistula, which drained the majority of the blood flow to the inferior petrosal sinus and only a small amount of blood flow to the ophthalmic vein. When the pressure in the ophthalmic vein increased, the venous blood flow was blocked, and the swollen vein gradually put pressure on the optic canal, resulting in eye distension and pain. As the pressure increased, the blood supply to the optic nerve was blocked, resulting in substantially decreased vision [6].

CCF can be categorized into four types based on the Barrow classification: cavernous sinus and ICA fistula (type A), ICA dural branch (type B), a meningeal branch of the external carotid artery (ECA) (type C), and both ICA and ECA meningeal branches (type D) [7]. This patient experienced a type B CCF combined with a DSA, and the fistula was located in the inferior pituitary artery. Very few patients develop bilateral symptoms for the bilateral cavernous sinus that communicates via the superior and inferior cavernous sinus [8].

The treatment of spontaneous CCF varies based on the different supply arteries that are involved, the location of the fistula, and the flow rate [9], and includes conservative treatment, endovascular treatment, microsurgical therapy and radiotherapy. Most patients exhibit a type B, C or D CCF, which are characterized by low blood flow, mild symptoms, a slow course and self-healing. Conservative treatment and carotid artery compression can be performed as the primary treatments for mildly affected patients, providing excellent results. Endovascular surgery is recommended when routine treatment is ineffective, significant cortical venous drainage occurs, or if vision declines precipitously [10]. This patient’s vision improved with glucocorticoid therapy, which reduced the ocular venous edema. Finger compression of the common carotid artery was the fundamental treatment for this patient, suggesting that other CCF patients with mild symptoms could be treated with this procedure early in the disease process.

## CONCLUSION

Spontaneous CCF is very rare and has a variety of clinical manifestations. This case is especially rare to have simple optic nerve involvement. Early diagnosis and etiological treatment can improve the prognosis.

## INFORMED CONSENT/INSTITUTIONAL APPROVAL

Informed consent was obtained from the patient to publish their case details and accompanying images. This case report did not require any institutional approval as per the IRB guidelines of our hospital.

## References

[1] Canellas M, Cheema N. Misdiagnosed spontaneous carotid cavernous sinus fistula. Clin Pract Cases Emerg Med 2019;3:256–8. 31404328 10.5811/cpcem.2019.4.42247 PMC6682224

[2] Adam CR, Shields CL, Gutman J, Kim HJ, Hayek B, Shore JW et al. Dilated superior ophthalmic vein: clinical and radiographic features of 113 cases. Ophthal Plast Reconstr Surg 2017;34:68–73. 10.1097/IOP.0000000000000872 28141624

[3] Griauzde J, Gemmete JJ, Pandey AS, Chaudhary N. Dural carotid cavernous fistulas: endovascular treatment and assessment of the correlation between clinical symptoms and the Cognard classification system. J Neurointerv Surg 2017;9:583–6. 27281178 10.1136/neurintsurg-2016-012418

[4] Fan Y, Yuechun LI, Zhang T, Jiang C, Zhang P. Carotid-cavernous sinus fistula caused by persistent primitive trigeminal artery aneurysm rupture: a case report. J Stroke Cerebrovasc Dis 2019;28:104306. 31439523 10.1016/j.jstrokecerebrovasdis.2019.104306

[5] Sirakov SS, Kitov BD, Sirakova KS, Kehayov II. Spontaneous direct carotid-cavernous fistula in an elderly patient. Folia Med (Plovdiv) 2017;59:472–6. 29341947 10.1515/folmed-2017-0059

[6] Oh DJ, Chhadva P, Kanu LN, Liu CY, MacIntosh P. Sudden-onset blindness from a spontaneous carotid-cavernous fistula with secondary central retinal artery occlusion and posterior ischemic optic neuropathy. Neuro-Ophthalmology 2018;43:107–13. 31312235 10.1080/01658107.2018.1488979 PMC6619923

[7] Leone G, Renieri L, Enriquez-Marul A, Dmytriw AA, Nappini S, Laiso A et al. Carotid cavernous fistulas and Dural arteriovenous fistulas of the cavernous sinus: validation of a new classification according to venous drainage. World Neurosurg 2019;128:e621–31. 31075494 10.1016/j.wneu.2019.04.220

[8] Sobin L, Jones K, Tatum S. Spontaneous carotid-cavernous fistula: challenges in clinical and radiologic diagnosis. Am J Emerg Med 2014;32:691.e3–5. 10.1016/j.ajem.2013.12.008 24656959

[9] Al-Mufti F, Amuluru K, El-Ghanem M, Changa AR, Singh IP, Gandhi CD et al. Spontaneous bilateral carotid-cavernous fistulas secondary to cavernous sinus thrombosis. Neurosurgery 2017;80:646–54. 28362925 10.1093/neuros/nyw128

[10] Iampreechakul P, Tirakotai W, Tanpun A, Wattanasen Y, Lertbusayanukul P, Siriwimonmas S. Spontaneous resolution of direct carotid-cavernous fistulas: case series and literature review. Interv Neuroradiol 2018;25:71–89. 30244626 10.1177/1591019918800220 PMC6378520

